# Early childhood caries, climate change and the sustainable development goal 13: a scoping review

**DOI:** 10.1186/s12903-024-04237-2

**Published:** 2024-05-03

**Authors:** Morẹ́nikẹ́ Oluwátóyìn Foláyan, Robert J Schroth, Olunike Abodunrin, Ola B. Al-Batayneh, Arheiam Arheiam, Tshepiso Mfolo, Jorma I. Virtanen, Duangporn Duangthip, Carlos A Feldens, Maha El Tantawi

**Affiliations:** 1 Early Childhood Caries Advocacy Group, University of Manitoba, Winnipeg, Canada; 2 Department of Child Dental Health, Obafemi Awolowo University, Ile-Ife, Nigeria; 3 Dr. Gerald Niznick College of Dentistry, University of Manitoba, Winnipeg, Canada; 4 Lagos State Health Management Agency, Lagos, Nigeria; 5 Department of Orthodontics, Pediatric and Community Dentistry, College of Dental Medicine, University of Sharjah, Sharjah, United Arab Emirates; 6 Department of Preventive Dentistry, Faculty of Dentistry, Jordan University of Science and Technology, Irbid, Jordan; 7 Department of Dental Public Health, Faculty of Dentistry, University of Benghazi, Benghazi, Libya; 8 Faculty of Health Sciences, School of Dentistry, Department of Community Dentistry, University of Pretoria, Pretoria, South Africa; 9 Faculty of Medicine, University of Bergen, Bergen, Norway; 10 College of Dentistry , The Ohio State University, Ohio Columbus, USA; 11 Department of Pediatric Dentistry, Universidade Luterana Do Brasil, Canoas, Brazil; 12 Department of Pediatric Dentistry and Dental Public Health, Faculty of Dentistry, Alexandria University, Alexandria, Egypt

**Keywords:** Climate change, Sustainable natural resource management, Human security, Carbon footprints, Dental treatment, Preventive dentistry, Pediatric dentistry

## Abstract

**Background:**

Sustainable development goal 13 centres on calls for urgent action to combat climate change and its impacts. The aim of this scoping review was to map the published literature for existing evidence on the association between the Sustainable Development Goal (SDG) 13 and early childhood caries (ECC).

**Methods:**

The scoping review followed the Preferred Reporting Items for Systematic Reviews and Meta-Analyses Extension for Scoping Reviews (PRISMA-ScR) guidelines. In August 2023, a search was conducted in PubMed, Web of Science, and Scopus using search terms related to SDG13 and ECC. Only English language publications were extracted. There was no restriction on the type of publications included in the study. A summary of studies that met the inclusion criteria was conducted highlighting the countries where the studies were conducted, the study designs employed, the journals (dental/non-dental) in which the studies were published, and the findings. In addition, the SDG13 indicators to which the study findings were linked was reported.

**Results:**

The initial search yielded 113 potential publications. After removing 57 duplicated papers, 56 publications underwent title and abstract screening, and two studies went through full paper review. Four additional papers were identified from websites and searching the references of the included studies. Two of the six retrieved articles were from India, and one was China, Japan, the United States, and the United Kingdom respectively. One paper was based on an intervention simulation study, two reported findings from archeologic populations and three papers that were commentaries/opinions. In addition, four studies were linked to SDG 13.1 and they suggested an increased risk for caries with climate change. Two studies were linked to SDG 13.2 and they suggested that the practice of pediatric dentistry contributes negatively to environmental degradation. One study provided evidence on caries prevention management strategies in children that can reduce environmental degradation.

**Conclusion:**

The evidence on the links between SDG13 and ECC suggests that climate change may increase the risk for caries, and the management of ECC may increase environmental degradation. However, there are caries prevention strategies that can reduce the negative impact of ECC management on the environment. Context specific and inter-disciplinary research is needed to generate evidence for mitigating the negative bidirectional relationships between SDG13 and ECC.

**Supplementary Information:**

The online version contains supplementary material available at 10.1186/s12903-024-04237-2.

## Introduction

Worldwide, the mean temperatures have climbed by approximately 1 °C (1.7 °F) since 1880, with projections indicating a potential warming of around 1.5 degrees Celsius (2.7 °F) by 2050 and a more substantial increase of 2–4 degrees Celsius (3.6–7.2 degrees Fahrenheit) by 2100 [1]. This alteration holds significance due to the immense heat energy required to elevate the earth’s average annual surface temperature, even by a slight margin, considering the vast extent and heat-retaining capacity of oceans. The approximately 2-degree Fahrenheit (1-degree Celsius) upswing in the global average surface temperature since the pre-industrial period (1880–1900) might appear modest, yet it equates to a substantial accumulation of heat [2].

Climate change is a huge concern for health, and its impact is felt globally. According to the World Meteorological Organization, greenhouse gas emissions are more than 50% higher now than in 1990 [3], and the World Health Organization (WHO) reports that global warming is causing long-lasting changes to the climate system that threatens irreversible consequences [4]. About 91% of geo-physical disasters are climate-related and they have tremendous human impact. Between 1998 and 2017, there were about 1.3 million deaths and 4.4 billion injuries due to the consequences of climate change [5].

The suggested health effects of climate change include changes in the prevalence and geographical distribution of respiratory diseases [6, 7]. Respiratory diseases such as asthma and its medicines increase the risk of caries [8–10]. The increase in greenhouse gas emissions and global warming are associated with an increase in geophysical disasters [11]. These disasters result in humanitarian crises [12] which likely increase the burden of dental disease, including early childhood caries (ECC) [13]. The emitted gases that deplete the ozone layer include methane and nitrous oxide emissions [14]. Methane is suggested to have an inverse association, and nitrous oxide has a direct association with global ECC prevalence [15].

Climate change is also associated with food insecurity, which is linked to caries [16, 17]. The risk of ECC may also increase with economic development, industrialization, and urbanization [18, 19], a phenomenon associated with increased gas emission and ozone depletion [20]. On the other hand, climate change also leads to arid conditions and high alkalinity in the ground waters, which promote fluoride release from clay and fluorite-bearing minerals [21, 22]. High temperatures promote longer residence times of ground waters, thereby leading to high fluoride contents of the water from water-rock interactions [21, 22]. Although fluorides in water are beneficial for dental health leading to reduced risk of ECC, excessive exposure to fluoride can result in severe fluorosis [23] which increases the risk of caries [24, 25].

The plausible link between climate change and ECC makes the Sustainable Development Goal 13 (SDG13) a subject of interest for pediatric dental care. The SDG 13 is focused on preventing and or tackling problems posed by climate change. It acknowledges that climate change is causing a rise in the occurrence and severity of extreme weather events, including floods, heatwaves, droughts, and tropical cyclones. These, in turn, heighten health risks due to damage to vital infrastructure, disruption of essential services like water and sanitation, education, energy, health, and transportation, exacerbation of water management challenges, and a decrease in agricultural output and food security [26–28]. These micro-, meso- and macro-level effects of climate change may increase the risk of ECC as it may cause disruption in access to preventive and curative care, limited access to health promotion, prevention information and education and increase the impact of food insecurity on ECC [29, 30].

**Fig. 1:**
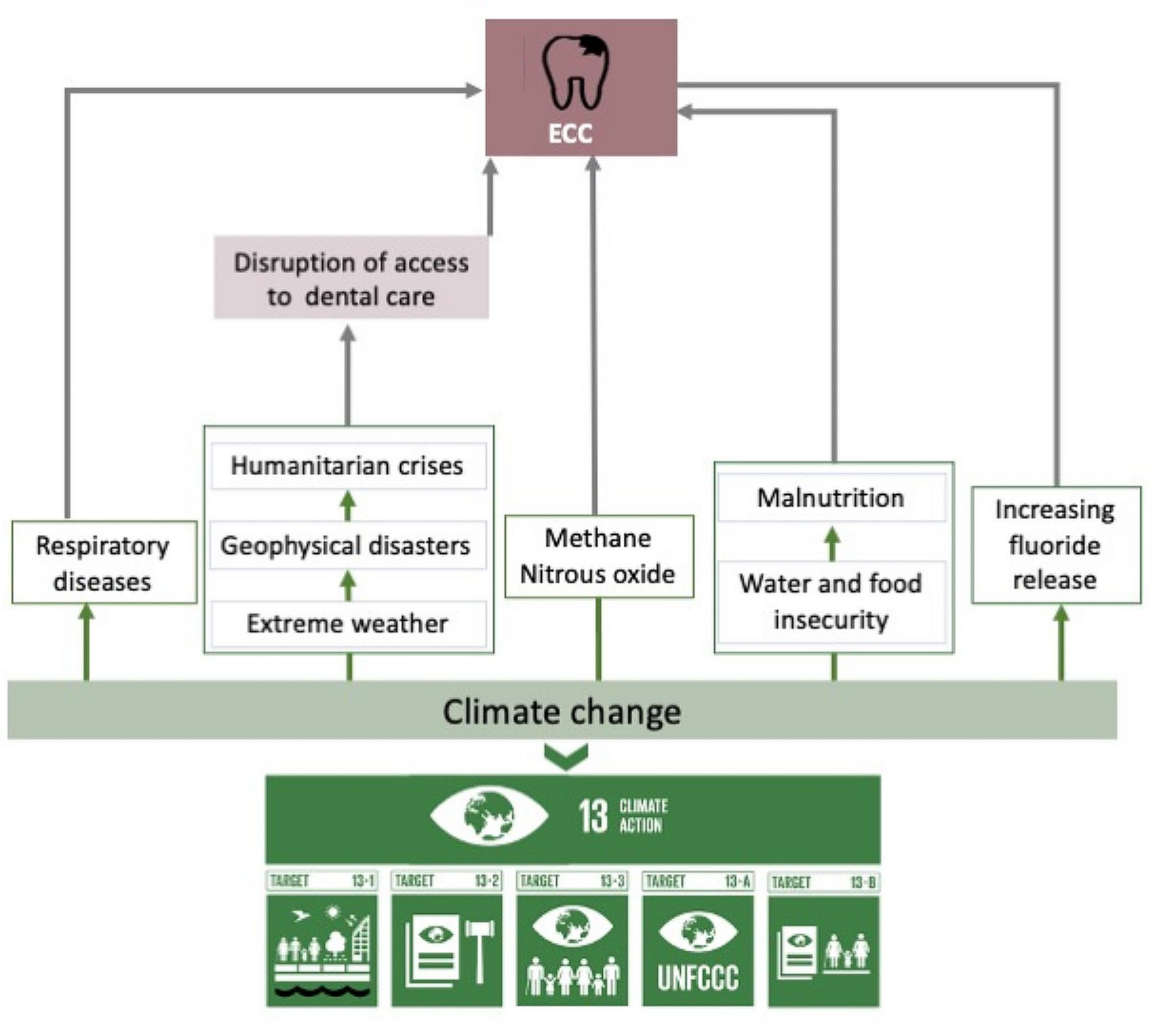
Conceptual framework for the relationship between climate change and ECC. Target 13.1 | Strengthen Resilience and Adaptive Capacity to Climate Related Disasters. Target 13.2 | Integrate Climate Change Measures into Policies and Planning. Target 13.3 | Build Knowledge and Capacity to Meet Climate Change.Target 13.A | Implement the UN Framework Convention on Climate Change. Target 13.B | Promote Mechanisms to Raise Capacity for Climate Planning and Management

A positive impact on ECC control may be linked with efforts at strengthening the resilience and adaptive capacity to climate-related hazards and natural disasters (SDG13.1); improving education, awareness and human and institutional capacity on climate change mitigation, adaptation, impact reduction and early warning (SDG 13.2); and integrating climate change measures into national policies, strategies, and planning (SDG 13.3) [26]. . Furthermore, incorporating the commitment made by developed-country parties to the United Nations Framework Convention on Climate Change, aimed at addressing the requirements of developing nations (13.A); and promoting mechanisms for raising capacity for effective climate change-related planning and management with focus on marginalized communities among others (SDG 13.B) [24], could also influence ECC control. This is because the prevalence of ECC is higher in developing countries [31] and among marginalized communities [32]. The conceptual framework for the association between climate change and ECC is presented in Fig. 1.

The aim of this scoping review was to identify the existing evidence on the association between climate change and climate change-related factors (disasters, sustainable management of natural resource, and human security) with ECC.

## Methods

We conducted a systematic search to identify scientific literature on the association between climate change and ECC. Our scoping review was conducted according to the JBI guidelines for scoping review [33] and reported in accordance with the Preferred Reporting Items for Systematic Reviews and Meta-Analyses Extension for Scoping Reviews guidelines (PRISMA-ScR) [34].

### Research questions

The following questions guided this review: (i) What is the existing evidence on the association between climate change and ECC? and (ii) What are the factors related to climate change (disasters, sustainable management of natural resource, human security) associated with ECC?”

### Articles identification

The initial search was conducted on three electronic databases (PubMed, Web of Science and Scopus) in August 2023. The search was performed using the key terms as shown in Appendix 1. Publications from the inception of each database to August 2023 were screened. No protocol was published for this review. Additional search was conducted by reviewing the references of eligible publications and by searching the semantics scholar website.

### Selection of articles

Article inclusion was performed in four phases. The first phase was conducted by one reviewer (MET) who conducted the search in the three databases for the information. In the second phase, two reviewers (OA, MOF) screened the titles and abstracts of all identified manuscripts and removed the duplicates. In phase three, two reviewers (OA, MOF) reviewed the full text of the manuscripts independently and compared results to achieve consensus. In addition, and reference lists of potentially relevant publications were manually searched. Lastly, the information generated was shared with two experts for their review (MET and RJS).

### Eligibility criteria

Articles were included if they focused on children younger than 6 year of age or if they did not specifically exclude this age group. In addition, studies that identified ECC as dependent or independent factors in relation to climate change or climate change related factors were included. No study design was excluded based on study design. There was no language restriction for the search conducted in the three databases. Language restrictions were introduced at the phase of review of the full texts. Articles not published in English were excluded. We also excluded studies that focused on the prevalence of ECC or on climate change exclusively.

### Data charting

Specific information from the included publications was extracted. This includes information on the first author’s name, year of publication, study location, World Health Organization’s region where the study was conducted (African (AFR), Eastern Mediterranean (EMR), European (EUR), Region of the Americas (AMR), South-East Asian (SEAR), and Western Pacific (WPR)) [35], study design, study objectives, main findings and conclusion on the association between SDG13 and ECC, and whether the article was published in dental or non-dental journal. Information from each publication was compiled and summarized in Table 1. The summarized data were then shared with two experts (RJS and AA) for their review. Publications were included only when there was a consensus between the experts and the earlier three reviewers. The final consensus document was also shared with members of the Early Childhood Caries Advocacy Group (www.eccag.org) to identify any other relevant publication that might not have been retrieved by the original search strategy.

### Data analysis

We performed a descriptive analysis of the extracted items. These descriptions encompassed the World Health Organization’s region and countries where the studies were conducted, the study designs employed, the journals (whether dental or non-dental) in which the studies were published, and the findings. Interpretive inductive analyses of the objectives and conclusions of the studies were also conducted. In addition, an analysis was conducted linking the study findings with an SDG13 indicator.

### Role of the funding source

The study was funded out-of-pocket. This had no role in the study design, data collection, analysis, decision to publish, or preparation of the manuscript.

## Results

**Fig. 2:**
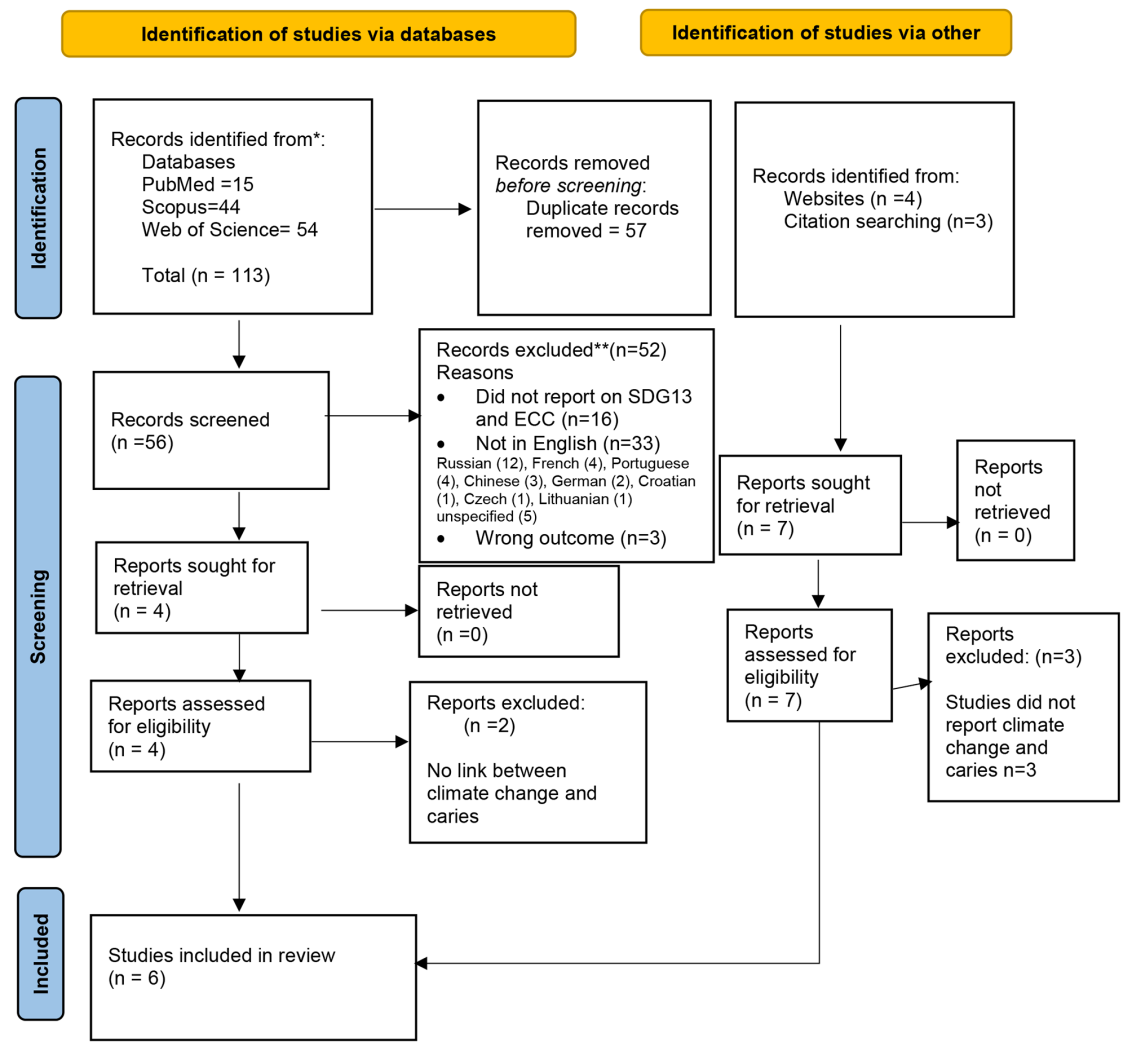
Flow diagram based on the Preferred Reporting Items for Systematic Reviews and Meta-Analyses 2020 flowchart template of the search and selected process

Figure 2 shows the process undertaken to identify relevant literature. The initial search of the three databases yielded 113 potential publications. Fifty-seven duplicated papers were removed, leaving 56 papers that underwent title and abstract screening. Of these, 54 papers were excluded leaving only two papers for study inclusion [36, 37]. In addition, two publications were identified from search in the semantic scholar website and another two identified by searching the references of one of the studies that met the eligibility criteria [36]. These additional four papers provided data on potential connections between climate change and caries [16, 38–40]. One of the six papers assessed the impact of management of ECC on climate change and ECC [37]. Table 1 presents further details regarding the six included publications.

**Table 1:** Characteristics of the studies included in the scoping review (*n* = 6)

Lead Author	Region (Location)	Year of publication	Journal type	Study design	SDG13 indicator link	Study objective	Conclusions
Acharya [36]	SEAR (India)	2023	Dental	Commentary/ opinion	13.2	To discuss the impact of climate change on paediatric dentistry and what changes can be made to provide environment-friendly solutions.	The use of environmentally harmful dental health-related materials (plastic toothbrushes and toothpaste, floss and mouthwash packaging, patient bags with toys and other items as gifts for children and single-use plastics where alternatives can be used) contribute detrimentally to environmental degradation, worsened health, health disparity and climate change.
Cheung et al. [37]	WPR (China)	2019	Non- dental	Archeological study	13.1	To examine published stable isotope and dental caries data of human skeletal remains from 77 archaeological sites across northern and northwestern China	The sudden change in subsistence economy across northern and northwestern China was likely related to climate change. The change in subsistence economy substantially increased the prevalence of dental caries from pre–to post–4000 BP due to increase in the consumption of cariogenic cereals during the later period.
Hackley [16]	AMR (USA)	2021	Dental	Commentary/ opinion	13.1	To expand the discussion of global climate disruption and to include considerations of oral health outcomes and dental practice crisis preparedness	Climate change is associated with heat stress and poor air quality, which were identified as risk factors for caries
Khanna [38]	SEAR (India)	2010	Non- dental	Commentary/ opinion	13.1	Not stated	Climatic variability and change also causes oxidative stress in allergic reactions that increases use of drugs for management of allergic disorders. These drugs could cause hypo-salivation or dry mouth, leading to increased incidence of dental caries
Lyne et al. [39]	EUR (United Kingdom)	2022	Dental	Intervention simulation study	13.2	To quantify the environmental impacts of fluoride varnish application in children using the life cycle impact assessment methodology	Fluoride varnish application in dental practice during an existing appointment had the lowest environmental impact followed by fluoride varnish application in schools. Fluoride varnish application at a separate dental practice appointment had the highest environmental impact with most of the impact resulting from the patient travel into dental practice.
Temple [40]	WPR (Japan)	2007	Non- dental	Archeological study	13.1	The dietary choices of the western/inland Jomon were assessed in relation to systemic stress by comparing carious tooth and enamel hypoplasia frequencies between the eastern and western/inland Jomon.	A subsistence shift during the Middle to Late Jomon period, perhaps in response to a changed climate, resulted in significant differences in carious tooth frequencies.

### Overview of studies

Two of the six retrieved articles were from India (SEAR) [36, 38], one was from China (the SEAR) [37], the US (AMR) [16], the UK (EUR) [39] and Japan (WRP) [40], respectively. The three papers from 2021 to 2023 were published in dental journals [16, 36, 39] while the three older papers were published between 2007 and 2019 in non-dental journals [37, 38, 40]. One paper was an intervention simulation study [39], two reported findings from archeologic populations [37, 40] and three papers that were commentaries/opinions [16, 36, 38].

The study objectives covered a range of topics from highlighting the need for environmentally-friendly solutions within dental practices to mitigate the ecological footprint of dental procedures [36], to the exploration of how environmental factors like climate may have influenced dental health in ancient populations [37, 40], and broader discussions on considerations and adaptation strategies to address the evolving environmental challenges of oral health management and outcomes in the context of global climate disruption [16, 39].

The study conclusions highlighted the interconnectedness between climate change, environmental sustainability, subsistence patterns, and oral health outcomes. It highlighted that the use of environmentally harmful dental materials contributes to environmental degradation and climate change [36]; climate change-induced shifts in subsistence economy, heat and oxidative stress, air quality that can significantly impact oral health outcomes [37] including increasing the risk for caries [16, 38, 39]. Alternative materials should be explored to mitigate these detrimental effects [36], and strategic approaches can be adopted to manage the impact of dental caries preventive on the climate [39].

In addition, the studies were linked to two targets of SDG13: specifically, SDG 13.1, which emphasizes enhancing resilience and adaptive capacity to climate-related hazards and natural disasters globally [16, 37, 38, 40]; and SDG 13.2, which focuses on integrating climate change measures into national policies, strategies, and planning [36, 39]. The four studies linking caries to SDG 13.1 suggests that adaptive measures to climate changes may increase the risk of caries. The two studies linking caries to SDG 13.2 indicate that pediatric dental practices have a negative impact on environmental degradation, consequently contributing adversely to climate change. However, one of the two studies proposes that specific actions can be taken to mitigate the environmental impact of caries prevention practices in children [39].

## Discussion

This scoping review identified articles that discussed how the discipline of pediatric dentistry’s carbon footprint can contribute to climate change. The studies identified a bi-directional relationship between caries and climate change. First, that adaptive processes for climate change can increase the prevalence of caries. Second, that the management of caries contributes to environmental degradation. Third, that purposeful strategic approaches to caries prevention in children can reduce the detrimental impact of caries management on the environment. These findings support our study hypothesis on the possible links between ECC and climate change.

However, the evidence supporting the study hypothesis are limited to commentaries, suggested archeological evidence and simulation studies. Studies on the impact of climate change are only starting to evolve. Methodological challenges may have limited the investigations into the link between climate change and ECC. Martens and McMichael identified a range of methodologies that could be used for studying the impact of climate change on health [42]. These methodologies are applicable for the study of the impact of climate change on oral health such as the evaluation of the impact of climate changes on shifts in the range and densities of caries predisposing organisms in the oral cavity, and children’s vulnerability to ECC. The current study identified an investigation that used implementation science approach to demonstrate how caries preventive practices in children can reduced the negative impact of dental practice on the environment [37]. It is therefore feasible to not only conduct studies to provide evidence on the link between ECC and climate change, but to demonstrate how the management of ECC can reduce environmental degradation. More studies are needed to identify context specific management strategies for ECC management that can be brought to scale.

One of the studies suggests a link between heat related climate changes and ECC [37]. We postulate that this link may result from the warming phenomenon that triggers an increase in water consumption. Existing evidence suggests that variations in water consumption levels between the coldest and warmest periods can fluctuate from 20% to as high as 60% [41]. Additionally, the intake of fluids per unit of body weight is most pronounced among infants and diminishes with advancing age [42]. Consequently, there may be a probable cumulative rise in children’s fluoride consumption due to global warming. This stems from multiple sources, including fluoride present in drinking water, fluoride-containing toothpaste, fluoride supplements, infant formula, beverages made with fluoridated water, cow’s milk from animals raised in fluoride-containing environments [43, 44], and crops cultivated in soil with elevated fluoride content due to interactions with water and rocks [17, 20]. The permissible threshold for fluoride intake is influenced by climatic conditions [45], with severely elevated fluoride intake, severe fluorosis, and a potential elevated risk of dental caries [22, 23, 46]. Geothermal temperature has been viewed as one cause for high fluoride levels recorded in groundwater (from deep aquifers and geothermal springs) [47]. On the other hand, Beltrán-Aguilar et al. demonstrated no association between outdoor temperature and the total water consumption of children aged 1 to 10 in the United States. This observation remained consistent even after accounting for age, gender, race/ethnicity, or poverty status [48]. . We, however, found no study addressing the link between outdoor temperature, consumption of water including fluoridated water and the prevalence of ECC. Future studies are needed to identify the pathophysiological pathways between climate change and ECC risks if there is truly a link.

It is also possible that the oxidative stress associated with climate change, arising from a disparity in the generation of pro-oxidant elements and the presence of antioxidant defenses [49] may be associated with enamel hypoplasia as highlighted by Temple [40]. Enamel hypoplasia may result from SOD1-mediated ROS accumulation disruption of normal enamel structure through alternative cervical loop cell proliferation and downregulation of RhoA and ROCK in ameloblasts [50]. Enamel hypoplasia is associated with an increased in the risk for ECC [51, 52].

There are, however, other possible pathophysiological pathways for the increased risk of ECC due to climate change not highlighted in the publications mapped in this scoping review. One plausibility may be linked to global warming that has the potential to induce pronounced aridification [53]. A temperature increases of 2 degrees Celsius would precipitate further arid conditions in 15% of semi-arid climates, potentially impacting over 25% of the globe [54]. This intensified aridification historically led to the desiccation of crops and increased dependence on marine resources for sustenance, akin to occurrences around 2000 B.C [55]. . This shift towards marine food sources is likely to encourage diets lacking in essential nutrients, potentially resulting in an increased rate of new bone formation on the outer surface of bones (periosteal new bone formation) [55]. However, there is a suggestion that carious lesions, premature tooth loss, and dental enamel hypoplasia might not necessarily experience an upswing due to aridification [55]. Studies on the pathways to link ECC and climate change are therefore, critically needed, to be able to take collective global actions to mitigate the negative oral health impact climate change may have on children.

Another pathophysiological pathway that may link climate change with an increase in the risk for ECC is the impact of rapid climate shifts on soil composition, solubility, and plant absorption [56]. These alterations could lead to a notable shift in the concentration of certain trace elements within plants, attributed to heightened translocation, improved photosynthetic capacity, and enhanced growth. Conversely, the warming process might result in reduced trace element concentrations in tubers, indicating that the tuber growth rate surpasses its ability to take in metals at elevated temperatures [57]. The presence of trace elements in plants contributes to the development of enamel and dentin [58], although the precise mechanisms governing the integration of trace elements into soils and, subsequently into human teeth require further elucidation [59]. The plausibleness of these interactions requires further investigations as the current study highlights that the objectives of the accessible studies on ECC and climate change are limited in the scope of their explorations.

In addition, the necessity for studies tailored to specific contexts highlights the limited regional coverage in current research examining the connections between ECC and climate change. Notably, our investigation revealed very few studies on this sub a lack of studies conducted in the AFR and EMR regions. Despite expectations that climate changes will alter rainfall patterns, impacting agriculture and diminishing food security while exacerbating water security issues in Africa [60], there is a notable absence of corresponding studies. Similarly, anticipated climate changes in the EMR region are expected to result in under-nutrition, respiratory illnesses, mental health issues, allergic reactions, and pulmonary diseases due to dust storms [61]. Despite being the two-worst impacted region in terms of health consequences resulting from climate change [62], these regions are currently underrepresented in evidence generation regarding the impact of climate change on health, including oral health. Consequently, significant gaps exist in our awareness and understanding of these links, potentially limiting mitigation and adaptation efforts [61]. It is, therefore, important to strengthen efforts to generate evidence from all regions with particular attention paid to AFR and EMR.

The suggested pathophysiological pathways for linking ECC and climate change clearing indicates the interconnectedness between climate change, environmental sustainability, subsistence patterns, and oral health outcomes. There is, therefore, the need for interdisciplinary and collaborative studies. One method that can be used to study the link between ECC and climate change is the integrated eco-epidemiologic models to identify the impact of climate change or stratospheric ozone depletion on the profile of organisms that cause caries; the thermal-related impact of climate change on the fluoride content of ground waters and its impact on caries risks; or the impact of ozone depletion on tooth structure and caries risk. These forms of study may present major scientific challenges in conceptualizing and technical difficulty with assessing the oral health impacts of these changes [42]. Eco-epidemiologic models will require a lot more anticipatory thinking and mathematical modelling of potential future impacts, which will be useful for policymakers [63].

Other study methods include the use of epidemiological surveillance techniques, assessment of the oral health impact of climate change using ecological frameworks, monitoring of the direct oral health impact of seasonal variations, natural disasters, marine ecosystems and ecosystems health, food production and food security, and emerging and resurgent infectious diseases [63]. Other methods include the use of retrospective study, integrated assessment modelling on oral health, and landscape epidemiology of caries profiles using remote censoring, Geographic Information system and spatial statistics [63]. The study methodologies, however, need to promote interdisciplinary research adapted for the modelling of complex processes and handling of attendant uncertainties [64].

The results confirmed the hypothesis regarding the connection between ECC and climate change, and mapping exercise highlighted areas where existing evidence has focused and identified new areas for further research. This is crucial not only for establishing links between ECC and all SDG13 indicators but, more importantly, for identifying ways to mitigate the negative bidirectional relationships between ECC and SDG13. Of interest are the archaeological studies identified in this study [37, 40]. These archaeological studies provide valuable insights into the historical and cultural determinants of caries, offering a unique perspective on the complex interplay between environmental, dietary, and sociocultural factors. By incorporating archaeological evidence into research and policy, stakeholders can advance efforts to address ECC and promote oral health within the framework of Sustainable Development Goal 13. Climate change is a major public health concern, and stakeholders need to proactively engage in mitigating the risk of poor oral health associated with poor climate controls using evidence that can be generated through multiple research strategies.

This scoping review, however, has a few limitations. First, our search was restricted to English literature only, potentially resulting in the omission of studies on the correlation between ECC and climate change published in other languages. This language restriction was solely applied during the article selection process for full-text review, ensuring transparency regarding the number of eligible reports available in languages other than English [65]. The decision to limit our search to English literature was made due to the inability to read and interpret literature written in other languages. Second, our search was limited to three data bases which may have led to the omission of relevant articles not captured by the search strategy, potentially introducing selection bias. The scope of the study is also limited to children under 6 years limiting the generalizability of findings to other age groups. Despite the limitations the study highlights plausible links between ECC and climate change that can be explored empirically in future studies.

In conclusion though there is the plausibility of climate change having an impact on the health of the dentition and the risk for caries. Studies are needed to generate empirical evidence of the impact of climate change on caries risk in children. This will help with the formulation of policies and the design of programs that can help policymakers and decision-makers proactively prevent the increase in the prevalence of ECC as we move into the future. Addressing these complex relationships is essential for developing holistic strategies to promote both environmental sustainability and oral health.

### Electronic supplementary material

Below is the link to the electronic supplementary material.

Supplementary Material 1

Supplementary Material 2

## Data Availability

No datasets were generated or analysed during the current study.

## Abbreviations

CINAHL: Cumulative Index to Nursing and Allied Health Literature
PRISMA: ScR-Preferred Reporting Items for Systematic Reviews and Meta-Analyses Extension for Scoping Reviews guidelines
