# Supplementary material for: Early childhood caries, climate change and the sustainable development goal 13: a scoping review
Source: BMC Oral Health. 2024 May 3;24:524. doi: 10.1186/s12903-024-04237-2 (PMC11067289; doi:10.1186/s12903-024-04237-2)
Supplement: Supplementary file 1 — Supplementary Material 1 [file 12903_2024_4237_MOESM1_ESM.docx]

**Appendix 1**

Search strategy for Scopus

Bottom of Form

Bottom of Form

Bottom of Form

| History Count | Search Terms | Results |
| --- | --- | --- |
| 3 | ( TITLE-ABS-KEY ( ( {climate action}  OR  {climate adaptation}  OR  {climate change}  OR  {climate capitalism}  OR  ipcc  OR  {climate effect}  OR  {climate equity}  OR  {climate feedback}  OR  {climate finance}  OR  {climate change financing}  OR  {climate forcing}  OR  {climate governance}  OR  {climate impact}  OR  {climate investment}  OR  {climate justice}  OR  {climate mitigation}  OR  {climate model}  OR  {climate models}  OR  {climate modeling}  OR  {climate modelling}  OR  {climate policy}  OR  {climate policies}  OR  {climate risk}  OR  {climate risks}  OR  {climate services}  OR  {climate service}  OR  {climate prediction}  OR  {climate predictions}  OR  {climate signal}  OR  {climate signals}  OR  {climate tipping point}  OR  {climate variation}  OR  {climate variations}  OR  ecoclimatology  OR  eco-climatology  OR  {Green Climate Fund}  OR  {regional climate}  OR  {regional climates}  OR  {urban climate}  OR  {urban climates}  OR  ( climate  AND  ( {adaptive management}  OR  awareness  OR  bioeconomy  OR  carbon  OR  {decision-making}  OR  {disaster risk reduction}  OR  {environmental education}  OR  {sustainable development education}  OR  {energy conservation}  OR  emission*  OR  extreme  OR  {food chain}  OR  {food chains}  OR  framework  OR  hazard*  OR  island*  OR  {land use}  OR  megacit*  OR  consumption  OR  production  OR  {small island developing states}  OR  anthropocene  OR  atmospher*  OR  {clean development mechanism}  OR  {glacier retreat}  OR  warming  OR  greenhouse  OR  {ice-ocean interaction}  OR  {ice-ocean interactions}  OR  {nitrogen cycle}  OR  {nitrogen cycles}  OR  {ocean acidification}  OR  {radiative forcing}  OR  {sea ice}  OR  {sea level}  OR  {sea levels}  OR  {thermal expansion}  OR  unfccc  OR  ozone ) ) )  AND NOT  ( {drug}  OR  {geomorphology} ) ) )  AND  ( TITLE-ABS-KEY ( caries )  OR  TITLE-ABS-KEY ( dental  AND  caries )  OR  TITLE-ABS-KEY ( dental  AND  decay )  OR  TITLE-ABS-KEY ( dental  AND  cavities )  OR  TITLE-ABS-KEY ( enamel  AND  demineralization )  OR  TITLE-ABS-KEY ( tooth  AND  demineralization )  OR  TITLE-ABS-KEY ( tooth  AND  cavities ) ) View Less | [44 document results](https://08105gjxz-1105-y-https-www-scopus-com.mplbci.ekb.eg/search/history/results.uri?origin=searchhistory&shid=4) |
| 2 | TITLE-ABS-KEY ( caries )  OR  TITLE-ABS-KEY ( dental  AND  caries )  OR  TITLE-ABS-KEY ( dental  AND  decay )  OR  TITLE-ABS-KEY ( dental  AND  cavities )  OR  TITLE-ABS-KEY ( enamel  AND  demineralization )  OR  TITLE-ABS-KEY ( tooth  AND  demineralization )  OR  TITLE-ABS-KEY ( tooth  AND  cavities ) | [125,423 document results](https://08105gjxz-1105-y-https-www-scopus-com.mplbci.ekb.eg/search/history/results.uri?origin=searchhistory&shid=3) |
| 1 | TITLE-ABS-KEY ( ( {climate action}  OR  {climate adaptation}  OR  {climate change}  OR  {climate capitalism}  OR  ipcc  OR  {climate effect}  OR  {climate equity}  OR  {climate feedback}  OR  {climate finance}  OR  {climate change financing}  OR  {climate forcing}  OR  {climate governance}  OR  {climate impact}  OR  {climate investment}  OR  {climate justice}  OR  {climate mitigation}  OR  {climate model}  OR  {climate models}  OR  {climate modeling}  OR  {climate modelling}  OR  {climate policy}  OR  {climate policies}  OR  {climate risk}  OR  {climate risks}  OR  {climate services}  OR  {climate service}  OR  {climate prediction}  OR  {climate predictions}  OR  {climate signal}  OR  {climate signals}  OR  {climate tipping point}  OR  {climate variation}  OR  {climate variations}  OR  ecoclimatology  OR  eco-climatology  OR  {Green Climate Fund}  OR  {regional climate}  OR  {regional climates}  OR  {urban climate}  OR  {urban climates}  OR  ( climate  AND  ( {adaptive management}  OR  awareness  OR  bioeconomy  OR  carbon  OR  {decision-making}  OR  {disaster risk reduction}  OR  {environmental education}  OR  {sustainable development education}  OR  {energy conservation}  OR  emission*  OR  extreme  OR  {food chain}  OR  {food chains}  OR  framework  OR  hazard*  OR  island*  OR  {land use}  OR  megacit*  OR  consumption  OR  production  OR  {small island developing states}  OR  anthropocene  OR  atmospher*  OR  {clean development mechanism}  OR  {glacier retreat}  OR  warming  OR  greenhouse  OR  {ice-ocean interaction}  OR  {ice-ocean interactions}  OR  {nitrogen cycle}  OR  {nitrogen cycles}  OR  {ocean acidification}  OR  {radiative forcing}  OR  {sea ice}  OR  {sea level}  OR  {sea levels}  OR  {thermal expansion}  OR  unfccc  OR  ozone ) ) )  AND NOT  ( {drug}  OR  {geomorphology} ) ) View Less | [688,992 document results](https://08105gjxz-1105-y-https-www-scopus-com.mplbci.ekb.eg/search/history/results.uri?origin=searchhistory&shid=2) |

Search strategy for WoS

- WOS.SCI: 1900 to 2023
- WOS.AHCI: 1975 to 2023
- WOS.BHCI: 2005 to 2023
- WOS.BSCI: 2005 to 2023
- WOS.ESCI: 2005 to 2023
- WOS.ISTP: 1990 to 2023
- WOS.SSCI: 1900 to 2023
- WOS.ISSHP: 1990 to 2023

| # | Search Query | Results |
| --- | --- | --- |
| 1 | (((((((((((((((((((((((((((((((((((((((TS=(climate action)) OR TS=(climate adaptation)) OR TS=(climate change)) OR TS=(climate capitalism)) OR TS=(ipcc )) OR TS=(climate effect)) OR TS=(climate equity)) OR TS=(climate feedback)) OR TS=(climate finance)) OR TS=(climate change financing)) OR TS=(climate forcing)) OR TS=(climate governance)) OR TS=(climate impact)) OR TS=(climate investment)) OR TS=(climate justice)) OR TS=(climate mitigation)) OR TS=(climate model)) OR TS=(climate models)) OR TS=(climate modeling)) OR TS=(climate modelling)) OR TS=(climate policy)) OR TS=(climate policies)) OR TS=(climate risk)) OR TS=(climate risks)) OR TS=(climate services)) OR TS=(climate service)) OR TS=(climate prediction)) OR TS=(climate predictions)) OR TS=(climate signal)) OR TS=(climate signals)) OR TS=(climate tipping point)) OR TS=(climate variation)) OR TS=(climate variations)) OR TS=(ecoclimatology )) OR TS=(eco-climatology )) OR TS=(Green Climate Fund)) OR TS=(regional climate)) OR TS=(regional climates)) OR TS=(urban climate)) OR TS=(urban climates) | 722836 |
| 2 | ((((((((((((((((((((((((((((((((((((((TS=(adaptive management)) OR TS=(awareness )) OR TS=(bioeconomy )) OR TS=(carbon )) OR TS=(decision-making)) OR TS=(disaster risk reduction)) OR TS=(environmental education)) OR TS=(sustainable development education)) OR TS=(energy conservation)) OR TS=(emission*)) OR TS=(extreme)) OR TS=(food chain)) OR TS=(food chains)) OR TS=(framework)) OR TS=(hazard*)) OR TS=(island*)) OR TS=(land use)) OR TS=(megacit*)) OR TS=(consumption)) OR TS=(production)) OR TS=(small island developing states)) OR TS=(anthropocene)) OR TS=(atmospher*)) OR TS=(clean development mechanism)) OR TS=(glacier retreat)) OR TS=(warming )) OR TS=(greenhouse)) OR TS=(ice-ocean interaction)) OR TS=(ice-ocean interactions)) OR TS=(nitrogen cycle)) OR TS=(nitrogen cycles)) OR TS=(ocean acidification)) OR TS=(radiative forcing)) OR TS=(sea ice)) OR TS=(sea level)) OR TS=(sea levels)) OR TS=(thermal expansion)) OR TS=(unfccc )) OR TS=(ozone ) | 11349140 |
| 3 | TS=(climate ) | 794359 |
| 4 | #3 AND #2 | 508542 |
| 5 | (TS=(drug)) OR TS=(geomorphology) | 2303768 |
| 6 | (#4) NOT #5 | 505258 |
| 7 | #6 OR #1 | 749796 |
| 8 | ((((((TS=(caries)) OR TS=(dental caries)) OR TS=(dental decay)) OR TS=(dental cavities)) OR TS=(tooth cavities)) OR TS=(tooth deminerali?ation)) OR TS=(enamel deminerali?ation) | 67592 |
| 9 | #7 AND #8 | 54 |

Search strategy for Pubmed

| Search number | Query | Results |
| --- | --- | --- |
| 10 | #8 AND #9 | 15 |
| 9 | (((((("Dental Caries"[Mesh]) OR "Tooth Demineralization"[Mesh]) OR (caries[Text Word])) OR (dental decay[Text Word])) OR (dental cavities [Text Word])) OR (tooth cavities[Text Word])) OR (enamel demineralization[Text Word]) | 71,670 |
| 8 | #1 OR #6 OR #7 | 89,034 |
| 7 | (climate change[MeSH Terms]) OR (Climate Models[MeSH Terms]) | 30,309 |
| 6 | #4 NOT #5 | 73,139 |
| 5 | (drug[Text Word]) OR (geomorphology[Text Word]) | 6,363,490 |
| 4 | #2 AND #3 | 74,927 |
| 3 | climate[Text Word] | 155,185 |
| 2 | ((((((((((((((((((((((((((((((((((((((adaptive management[Text Word]) OR (awareness[Text Word])) OR (bioeconomy[Text Word])) OR (carbon[Text Word])) OR (decision-making[Text Word])) OR (disaster risk reduction[Text Word])) OR (environmental education[Text Word])) OR (sustainable development education[Text Word])) OR (energy conservation[Text Word])) OR (emission*[Text Word])) OR (extreme[Text Word])) OR (food chain[Text Word])) OR (food chains[Text Word])) OR (framework[Text Word])) OR (hazard*[Text Word])) OR (island*[Text Word])) OR (land use[Text Word])) OR (megacit*[Text Word])) OR (consumption[Text Word])) OR (production[Text Word])) OR (small island developing states[Text Word])) OR (anthropocene[Text Word])) OR (atmospher*[Text Word])) OR (clean development mechanism[Text Word])) OR (glacier retreat[Text Word])) OR (warming[Text Word])) OR (greenhouse[Text Word])) OR (ice-ocean interaction[Text Word])) OR (ice-ocean interactions[Text Word])) OR (nitrogen cycle[Text Word])) OR (nitrogen cycles[Text Word])) OR (ocean acidification[Text Word])) OR (radiative forcing[Text Word])) OR (sea ice[Text Word])) OR (sea level[Text Word])) OR (sea levels[Text Word])) OR (thermal expansion[Text Word])) OR (unfccc[Text Word])) OR (ozone[Text Word]) | 3,911,219 |
| 1 | ((((((((((((((((((((((((((((((((((((climate action[Text Word]) OR (climate adaptation[Text Word])) OR (climate capitalism[Text Word])) OR (ipcc[Text Word])) OR (climate effect[Text Word])) OR (climate equity[Text Word])) OR (climate feedback[Text Word])) OR (climate finance[Text Word])) OR (climate change financing[Text Word])) OR (climate forcing[Text Word])) OR (climate governance[Text Word])) OR (climate impact[Text Word])) OR (climate investment[Text Word])) OR (climate justice[Text Word])) OR (climate mitigation[Text Word])) OR (climate modeling[Text Word])) OR (climate modelling[Text Word])) OR (climate policy[Text Word])) OR (climate policies[Text Word])) OR (climate risk[Text Word])) OR (climate risks[Text Word])) OR (climate services[Text Word])) OR (climate service[Text Word])) OR (climate prediction[Text Word])) OR (climate predictions[Text Word])) OR (climate signal[Text Word])) OR (climate signals[Text Word])) OR (climate tipping point[Text Word])) OR (climate variation[Text Word])) OR (climate variations[Text Word])) OR (ecoclimatology[Text Word])) OR (eco-climatology[Text Word])) OR (Green Climate Fund[Text Word])) OR (regional climate[Text Word])) OR (regional climates[Text Word])) OR (urban climate[Text Word])) OR (urban climates[Text Word]) | 10,636 |
